# *Coriolopsis strumosa* as an Orchid Endophytic Fungus and Its Spatial Distribution in *Epidendrum* sp. (Orchidaceae)

**DOI:** 10.3390/microorganisms12061054

**Published:** 2024-05-24

**Authors:** Na Yao, Tao Wang, Jingwan Jiang, Yuqian Yang, Xiaolu Cao

**Affiliations:** 1 State Key Laboratory of Tree Genetics and Breeding, Key Laboratory of Tree Breeding and Cultivation of the National Forestry and Grassland Administration, Research Institute of Forestry, Chinese Academy of Forestry, Beijing 100091, China; nayao@caf.ac.cn; 2 Beijing Botanical Garden, Beijing Floriculture Engineering Technology Research Centre, Key Laboratory of National Forestry and Grassland Administration on Plant Ex Situ Conservation, Beijing 100093, China; wangtao@chnbg.cn (T.W.); sunayc@163.com (J.J.); 15101178950@163.com (Y.Y.)

**Keywords:** plant endophytes, symbiosis, Polyporaceae, germination

## Abstract

*Coriolopsis* spp. are wood-decaying fungi that inhabit forests. They are mainly distributed in tropical and subtropical areas. Strain Epi910 was isolated from the asymbiotically germinated protocorm of *Epidendrum* sp. and identified as *Coriolopsis strumosa*. Symbiotic germination and high-throughput sequencing of the endophytic fungal communities of different parts were performed to characterize the function and spatial distribution of the Epi910 isolate. Under symbiotic germination, Epi910 promoted seed germination and seedling formation as an endophytic native fungus of *Epidendrum* sp. Endophytic fungal communities from seven different parts of *Epidendrum* sp. were characterized. In total, 645 OTUs were identified; 30 OTUs were shared among all seven parts. The internal transcribed spacer sequence of Epi910 was identical to that of a dominant shared OTU (OTU6). The relative abundance of OTU6 in the seven parts was identified as follows: capsule pericarp > seed > root > asymbiotically germinated protocorm > epiphytic root > ovary > rachis. Our results suggest that the isolate belonging to *Coriolopsis strumosa* could promote the germination of *Epidendrum* sp. There may, therefore, be endophytic fungi other than common orchid mycorrhizal fungi with the ability to enhance germination in orchids.

## 1. Introduction

Fungi are indispensable in the life history of orchids. By establishing a symbiotic relationship with orchid mycorrhizal fungi (OMF), orchids can acquire organic nutrients and carbon energy at life-history stages [1,2]. Most green orchids interact with rhizoctonia-like fungi, polyphyletic aggregates of species belonging to the Ceratobasidiaceae, Sebacinales, or Tulasnellaceae families [3,4,5]. These can colonize the cells of the protocorm or the root, forming intracellular hyphae coils [6,7]. Many other endophytic fungi have been isolated and their positive effects on the seed germination of orchids have been confirmed. Examples include species in Auriculariaceae [8], Thelephoraceae [9], Tricholomataceae [9], and Polyporaceae [10]. Fungi belonging to Polyporaceae such as *Loweporus tephroporus*, *Microporus affinis*, *Lenzites betulinus*, and *Trametes hirsuta* have been demonstrated to enhance the seed germination of orchids [10,11].

The genus *Coriolopsis* Murill (1905) (order Polyporales, phylum Basidiomycota) includes species that cause white rot in wood [12,13]. It has attracted considerable attention because of its capacity to degrade lignocellulosic materials [14,15]. As endophytic fungi, *Coriolopsis* spp. have been isolated from the roots of *Paphiopedilum villosum* (Lindl.) Stein [16] and *Cymbidium faberi* Rolfe [17]. Until now, it was uncertain whether *Coriolopsis* spp. could form intracellular hyphae and enhance seedling formation.

High-throughput sequencing has been used for microbiota analyses and has comprehensively revealed the temporal and spatial distribution of endophytic fungi in orchids [18,19]. Although the recruitment of fungal communities during the germination of orchid seeds has been studied [20], the composition of seed endophytic communities and their functions remain largely unexplored in orchids. This is in contrast to the research efforts assigned to rice [21], oak [22], and other plants, which focus on vertically transmitted microorganisms between generations and the potential functions of microorganisms.

*Epidendrum* L. is widely distributed in Central and South America. Certain species or hybrids belonging to the *Epidendrum* genus are used as ornamental plants because of the rich variations in the flower colors and the long flowering periods. Flowers of varying colors—including purple, pink, white, yellow, and red—grow in clusters on long flower spikes. These are commonly used for wedding decorations and flower arrangements [23]. OMF belonging to *Tulasnella*, *Ceratobasidium*, or *Sebacina* [24,25,26,27] can colonize the roots of *Epidendrum* sp. and establish a symbiotic relationship. *Peniophora incarnata*, a white-rot fungus, has been isolated from *Epidendrum* sp. and identified as a germination-enhancing fungus [28].

In this study, we isolated the fungal strain Epi910 from the germinated protocorm of *Epidendrum* sp. Symbiotic germinations were performed to characterize the function of Epi910 on the germination-enhancing properties of *Epidendrum* sp. High-throughput sequencing of the ITS region was used to characterize the fungal communities present in the seeds and pericarp of fruits as well as the roots, aseptically germinated protocorms, and floral organs. The objectives of this study were to identify Epi910 isolated from the asymbiotically germinated protocorm of *Epidendrum* sp., to investigate the effects of Epi910 on the in vitro germination of *Epidendrum* sp., and to demonstrate the spatial distribution of the Epi910 isolate colonized in different parts of *Epidendrum* sp.

## 2. Materials and Methods

### 2.1. Fungal Isolation and Identification

Seeds in a Petri dish germinated faster than those in other dishes during the asymbiotic germination of *Epidendrum* sp. on an oatmeal agar (OMA) medium (1.00 g oatmeal (Solarbio, Beijing, China) was added to 800 mL distilled water, boiled for 30 min, and filtered through a 50 mesh sieve to remove large particles; 6.00 g agar was then added and the sample volume was increased to 1000 mL with water). A fungal strain isolated from a protocorm germinated in advance was identified as Epi910. This was isolated and purified on potato dextrose agar (PDA) (Difco, BD, Franklin Lakes, NJ, USA) using the tissue section method after inspecting the colonization of the fungi in the protocorm with hand sectioning and histological observations [29]. A purified fungal isolate was obtained after the fungal mycelia were subcultured 3–4 times. The Epi910 isolate was preserved at 4 °C in vials with PDA at the Research Institute of Forestry, Chinese Academy of Forestry, and the China General Microbiological Culture Collection Center (CGMCC No. 21081). Two *Coriolopsis strumosa* strains (53709 and 53727) were procured from the China Forestry Culture Collection Center (CFCC); these were used as the reference strains in phylogenetic analysis.

The nuclear condition of young hyphae was observed after staining with SYBR Green I in accordance with Meinhardt’s method [30]. The observations, measurements, and photographic images of the microscopic fungal structures were recorded using a ZEISS Axio Imager M2 microscope (ZEISS, Oberkochen, Germany) with a ZEISS Axiocam 503 monocamera and differential interference contrast (DIC) illumination. ZEISS Zen 3.3 was used for the image processing and synthesis.

A CTAB rapid fungi genome extraction kit (E.Z.N.A.^®^ Fungal DNA Kit, Omega Bio-tek, Inc., Norcross, GA, USA) was used to extract total genomic DNA from the mycelia. Polymerase chain reactions (PCRs) were then performed. The internal transcribed spacer (ITS) region was amplified using the ITS1 and ITS4 primer pair [31]. The large subunit of the nuclear ribosomal DNA (nLSU) region was amplified using the LR0R and LR7 primer pair. The nSSU region was amplified using the PNS1 and NS41 primer pair [32]. The mtSSU region was amplified using the MS1 and MS2 primer pair [31]. Part of *EF1*-*α* was amplified using the EF1-983F and EF1-1567R primer pair [33]. *RPB1* was amplified using the RPB1-Af and RPB1-Cr primer pair [34]. *RPB2* was amplified using the fRPB2-5F and fRPB2-7cR primer pair [35]. PCR procedures were performed in accordance with the method of Ji et al. [36]. PCR products were purified and sequenced at a sequencing laboratory in Beijing (Sangon Biotech Co., Ltd., Shanghai, China) using the same primers. The sequences were Blasted against the GenBank database (National Center for Biotechnology Information) for identification and then deposited therein (accession numbers MT787240, MT893343, MT893345, MT895506, MT900754, MT905061, and MT951981).

A phylogenetic tree was generated using the ITS+nLSU sequences of each of the isolates obtained from the BLAST searches to visualize the phylogenetic position of Epi910 from *Epidendrum* sp. against other known *Coriolopsis* spp. associated with forest wood.

All sequences were aligned using MAFFT v7.311 [37] and manually adjusted using Mega X [38]. The gene fragments were spliced using Mesquite v3.3 [39] for the subsequent phylogenetic analyses. Sequences from *Boletopsis leucomelaena* (DQ484064 and DQ154112) were used as outgroups to root the tree [14]. Mega X was used to construct a maximum likelihood (ML) tree for the GTR+G+I model with 1000 bootstrap replicates. MrModeltest v2.3 [40,41] was used to determine the best-fit evolution model for the combined dataset for Bayesian inference (BI). BI was calculated using MrBayes v3.2.7 [42], with a general time-reversible (GTR+I+G) model of DNA substitution and a gamma distribution rate variation across the sites. Four Markov chains were processed for two runs from random starting trees for 1 million generations and sampled every 100 generations. The phylogenetic trees were visualized using FigTree v1.4.2. Branches that received bootstrap support for ML and Bayesian posterior probabilities greater than or equal to 50% and 0.70, respectively, were considered to be significantly supported.

### 2.2. Testing the Fungal Promotion of Seed Germination

The capacity of Epi910 to promote germination was tested using the seeds of *Epidendrum* sp. *Coriolopsis strumosa* (strain 53727) was used for the symbiotic germination to verify the capacity of the other *Coriolopsis strumosa* isolate to enhance the germination of *Epidendrum* sp.

In vitro seed germination was performed after the surface sterilization of fruit coats (30 s submergence in 75% ethanol followed by a 30 s rinse step using sterile distilled water, then 3 min submergence in 1% sodium hypochlorite followed by five 30 s rinse steps using sterile distilled water). Approximately 300–500 seeds were sown per Petri dish on an OMA medium. A fungal inoculum was placed in the center of each Petri dish and co-inoculated with seeds. Asymbiotic germinations were performed as controls on two media: OMA and MS1 (an MS medium with one-quarter-strength MS macronutrients, 0.40 mg/L 6-benzylaminopurine, 10.00 g/L sucrose, and 6.00 g/L agar; pH 6.30) [43]. Each treatment had three Petri dish replicates; these were placed in a germination chamber under 25 ± 2 °C and 16/8 h light/dark cycle conditions. The number of seeds and the status of seed germination were assessed 90 days after incubation for each Petri dish in accordance with the stages defined by Arditti [44]. The categorization was as follows: 0, no germination; 1, the embryo had swollen and turned green and the testa was propped up (germination); 2, continued embryo enlargement, with the formation of a spherule and a broken seed coat (protocorm formation); 3, the appearance of protomeristem (protocorm differentiation); 4, advanced seedlings, with the emergence of the first leaf; and 5, the emergence of the second leaf and further development. The percentages of germinated seeds at each developmental stage were calculated. Seed germination data were recorded in accordance with the method of Meng et al. [45]: 0, 1, (2 + 3), and (4 + 5) stages were defined based on the number of ungerminated and germinated (g) seeds, protocorms (p), and seedlings (s). The total number of seeds (t) was the sum of the number of seeds with well-developed embryos at each stage. The germination rate (G) and seedling formation rate (S) were calculated 90 days after inoculation using the following equations:

$$G=\left(g+p+s\right)÷t\times 100\;S=\left(s÷t\right)\times 100$$

Analysis of variance was performed using SPSS 16.0 (SPSS Inc., Chicago, IL, USA). Data were analyzed using a one-way analysis of variance (ANOVA) after an inverse sine transformation. Statistical significance was set as *p* < 0.05. The means of the samples were compared using the least significant difference (LSD) test.

### 2.3. Assessment of Endophytic Fungal Communities in Epidendrum sp.

Six different parts (root, epiphytic root, rachis, ovary, uncracked capsule pericarp, and seed) were collected from June to August 2020 to assess the endophytic fungal communities in different *Epidendrum* sp. organs. After the samples were carefully collected from the plants, they were transported to a laboratory and immediately surface-sterilized (30 s submergence in 75% ethanol followed by a 30 s rinse step using sterile distilled water, then 5 min submergence in 1% sodium hypochlorite with a vortex to remove any tightly adhering microbes followed by five 30 s rinse steps using sterile distilled water).

The in vitro asymbiotic germination of seeds was performed using an MS1 medium after the surface sterilization of uncracked capsules, as described in Section 2.2. Protocorms at stage 2–3 after 28–35 days of in vitro culture were harvested for an endophytic fungal community assessment.

Each of the samples were from five different individual plants with three replicates. The same sterilized sample from different plants was cut into 1–2 mm slices, mixed equally by weight, separately placed in sterile tubes according to the replicate, and stored at −80 °C until the DNA was extracted.

DNA was extracted from 500 mg samples using a QIAamp DNA Stool Mini Kit (QIAGEN, Hilden, Germany) in accordance with the manufacturer’s instructions. Amplicon libraries were created using general ITS primers (ITS1F 5′-CTTGGTCATTTAGAGGAAGTAA-3′ and ITS2R 5′-GCTGCGTTCTTCATCGATGC-3′) [46]. Overhang adapters were added to the primers to ensure compatibility with the Nextera Index Kit (Illumina, San Diego, CA, USA). Two sets of PCRs were produced. The initial PCRs were produced using 25 μL reaction volumes with 1–2 μL DNA template, 250 mM dNTPs, 0.25 mM of each primer, 1× reaction buffer, and 0.50 U Phusion DNA Polymerase (New England Biolabs, Ipswich, MA, USA). The PCR conditions comprised initial denaturation at 94 °C for 2 min followed by 35 cycles of denaturation at 94 °C for 30 s, annealing at 50 °C for 30 s, an extension at 72 °C for 30 s, and a final extension of 72 °C for 5 min. The second set of PCRs with dual eight-base barcodes were used for multiplexing. Eight-cycle PCRs were used to incorporate two unique barcodes at either end of the ITS amplicons. The cycling conditions consisted of one cycle at 94 °C for 3 min followed by eight cycles at 94 °C for 30 s, 56 °C for 30 s, and 72 °C for 30 s, with a final extension cycle at 72 °C for 5 min. Purified amplicons were pooled for each sample in equimolar concentrations and used for paired-end sequencing on an Illumina MiSeq PE300 platform (Illumina, San Diego, CA, USA) in accordance with the standard protocols of TinyGene Bio-Tech Co., Ltd. (Shanghai, China).

Raw fastq files were demultiplexed based on the barcode. PE reads for all samples were run through Trimmomatic v0.35 [47] to remove low-quality base pairs using the parameters SLIDINGWINDOW: 50:20 and MINLEN: 50. FLASH v1.2.11 was then used to merge the trimmed reads with the default parameters. Low-quality contigs were removed using the screen.seqs command based on the following filtering parameters: maxambig = 0, minlength = 200, maxlength = 580, and maxhomop = 8. ITS fragments were analyzed using a combination of Mothur v1.33.3 [48], UPARSE, and R v3.2.3. Demultiplexed reads were clustered at a sequence identity of 97% into operational taxonomic units (OTUs) using the UPARSE pipeline. Representative OTU sequences were taxonomically assigned to the UNITE v8 database with a confidence score ≥ 0.80 using the classify.seqs command in Mothur v1.33.3. Beta-diversity analysis of the hierarchical cluster diagram was ascertained using R v3.6.0. Raw reads were deposited into the NCBI Sequence Read Archive Database (accession number PRJNA754791).

## 3. Results

### 3.1. Morphological and Molecular Identification of the Epi910 Isolate

**Figure 1:**
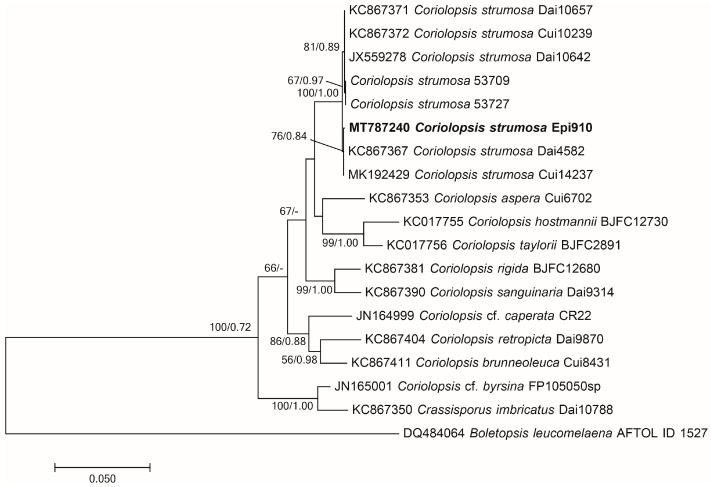
Phylogenetic tree of *Coriolopsis strumosa* Epi910 (in bold) and related fungi based on maximum likelihood (ML) and MrBayes analyses of ITS+nLSU sequences. Branches were labeled based on an ML bootstrap > 50% and Bayesian posterior probabilities > 0.70. *Boletopsis leucomelaena* (AFTOL ID 1527) was used as the outgroup. ITS: internal transcribed spacer; nLSU: large subunit of nuclear ribosomal DNA.

**Figure 2:**
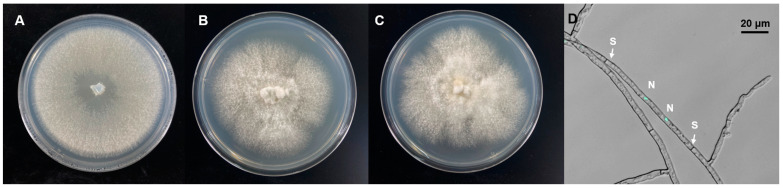
Colony and fungal hyphae morphology of *Coriolopsis strumosa.* Colony morphology of three *C. strumosa* isolates on a potato dextrose agar (PDA) medium: (**A**) Epi910; (**B**) 53709; (**C**) 53727. (**D**) Hyphae of Epi910 stained with SYBR Green I revealing binucleate cells (N: nuclei; S: septa).

Molecular identification and phylogenetic analysis revealed that the Epi910 isolate belonged to *Coriolopsis strumosa* (Figure 1). The colony morphology of Epi910 on the PDA medium was white to pale cream with regular, submersed edges and dense aerial mycelia (Figure 2A). The hyphae were hyaline with binucleate cells (Figure 2D).

### 3.2. Fungal Capacity to Enhance Seed Germination

**Figure 3:**
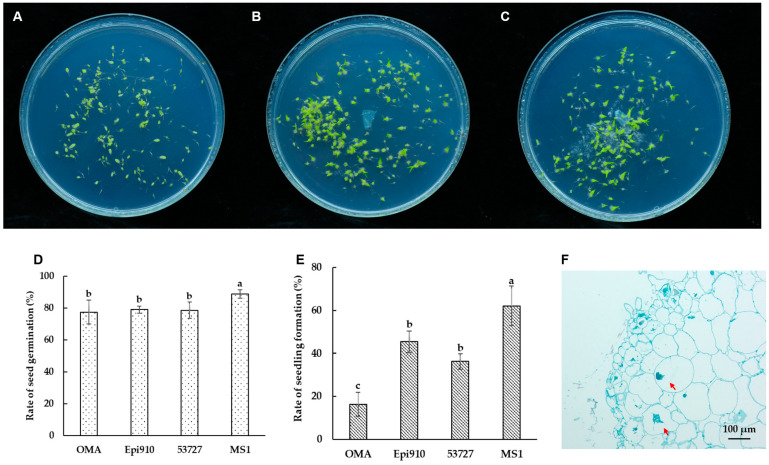
In vitro symbiotic germination of *Epidendrum* sp. with two *Coriolopsis strumosa* isolates (Epi910 and 53727). (**A**) In vitro seed germination on an OMA medium in 90 mm Petri dishes as a control without fungi. (**B**,**C**) In vitro symbiotic germination of seeds with fungal isolates Epi910 (**B**) and 53727 (**C**) on an OMA medium. (**D**,**E**) Rates of seed germination (**D**) and seedling formation (**E**) after 90 days of germination. (**F**) Epi910 colonized the inner cells of protocorms and formed intracellular hyphae coils after in vitro symbiotic germination. Different lowercase letters represent significant differences (*p* ˂ 0.05). The red arrow indicates the intracellular fungal peloton.

Epi910 and 53727 promoted the germination of *Epidendrum* sp. in the in vitro symbiotic germination experiment. After 90 days of incubation, significant differences were observed between symbiotic germination and asymbiotic germination on the OMA medium. This observation was based on the percentages of advanced seedling development in *Epidendrum* sp. (Figure 3A–C).

Over 88% seed germination of *Epidendrum* sp. was observed in the nutrient-rich treatment (MS1). This resulted in large proportions of advanced seedlings (62.09%; Figure 3D,E). Only 16.23% of the seeds formed advanced seedlings in the nutrient-poor (OMA) treatment. Epi910 and 53727 colonized *Epidendrum* sp. seeds, promoting seedling formation. This was significantly higher than the seedling formation percentage in the nutrient-poor (OMA) treatment and lower than that of the nutrient-rich (MS1) treatment.

After 90 days of inoculation with *Coriolopsis strumosa*, the fungal hyphae colonized the protocorm cells and formed intracellular pelotons in *Epidendrum* sp. (Figure 3F).

### 3.3. Community Structure of Endophytic Fungi in Epidendrum sp.

**Figure 4:**
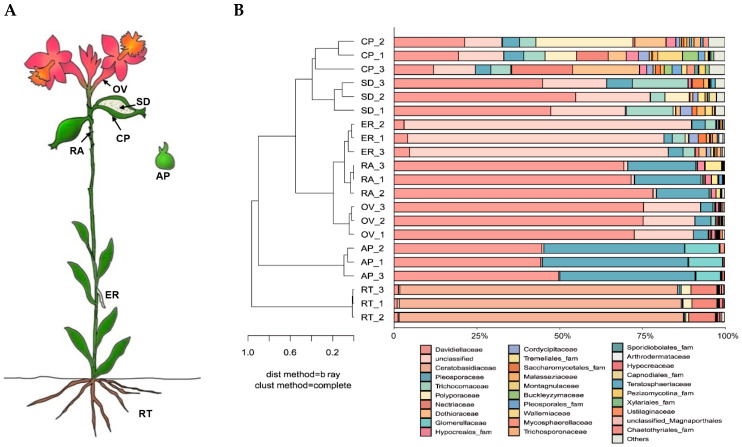
Community structure of endophytic fungi in *Epidendrum* sp. (**A**) Sampling positions of *Epidendrum* sp. (RT: root; ER: epiphytic root; RA: rachis; OV: ovary; CP: capsule pericarp; SD: seed; AP: asymbiotically germinated protocorm). (**B**) Classification and relative abundance of fungal endophytes at the family level for all seven parts.

The endophytic fungal communities associated with seven *Epidendrum* sp. parts (Figure 4A) were detected via the high-throughput sequencing of the ITS. The quality-filtered sequencing dataset comprised 618,240 sequences. Of these, 576,878 sequences (93.31%) were assigned to 645 OTUs belonging to 19 classes, 55 orders, 103 families, and 166 genera. The endophytes in the roots were dominated by fungi belonging to the phylum Basidiomycota (87.81%), whereas the protocorms germinated in vitro were dominated by unclassified fungi (80.44%). Other parts were dominated by fungi from the phylum Ascomycota.

Fungal community compositions were significantly different among the seven orchid parts at the family level. With the exception of the root, the endophytic fungal communities of the other six samples were clustered into a large group at the family level. The seed and capsule pericarp were grouped together, whereas the epiphytic root, ovary, rachis, and asymbiotically germinated protocorm were grouped together (Figure 4B). This indicated that the distribution of endophytic fungal communities in *Epidendrum* sp. had a positional effect. The endophytic fungal community structure of the root as an underground part was significantly different from the aboveground parts. The dominant fungal groups in the root were Ceratobasidiaceae (85.03%), Nectriaceae (7.84%), Polyporaceae (2.16%), and Davidiellaceae (1.19%). The dominant fungal groups in the asymbiotically germinated protocorm were unclassified fungi (80.82%), Davidiellaceae (3.96%), Pleosporaceae (3.68%), Trichocomaceae (3.55%), and Cordycipitaceae (1.55%).

### 3.4. Spatial Distribution of the Shared Endophytic Fungi

**Figure 5:**
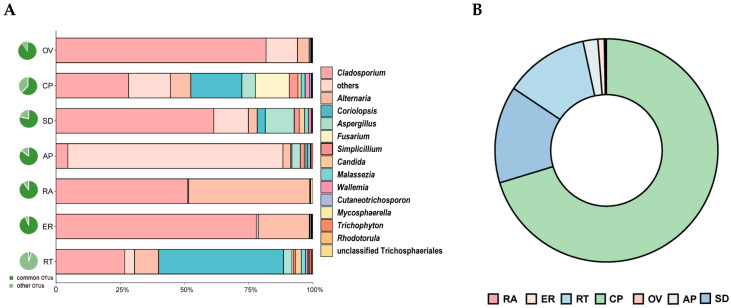
Relative abundance of shared OTUs. (**A**) Proportion of shared OTUs among all OTUs in corresponding samples (pie chart on the left of Figure 5A) and relative abundance of shared OTUs by genera in all seven samples (right). (**B**) Relative abundance of OTU6 (*C. strumosa*) in all seven samples.

Thirty OTUs belonging to fifteen genera were detected across all seven samples. The shared OTUs exhibited different abundance patterns among the samples. Excluding the unclassified OTUs, *Cladosporium*, *Alternaria*, and *Coriolopsis* were the dominant groups in the total abundance of shared genera (Figure 5A; Table S1). The relative abundance of *Cladosporium* was higher in the epiphytic root, seed, and ovary. Conversely, the relative abundance of *Alternaria* was higher in the rachis, epiphytic root, and root. *Coriolopsis* had a higher abundance in the root, capsule pericarp, and seed (Figure 5A).

OTU6, which was shared among all seven parts, was identified in *Coriolopsis* and was 100% identical to the ITS of Epi910. This OTU exhibited a high relative abundance in the capsule pericarp and seed but a low relative abundance in the ovary and rachis. Relative abundance was identified in the following order: capsule pericarp > seed > root > asymbiotically germinated protocorm > epiphytic root > ovary > rachis (Figure 5B, Table S2).

## 4. Discussion

The fungi used for symbiotic germination are mainly isolated from the root or the protocorm of orchids germinated in situ [49,50]. In our study, a germination-enhancing fungus (Epi910) belonging to a species of *Coriolopsis strumosa* was isolated from the in vitro asymbiotic germinated protocorm of *Epidendrum* sp. Based on an analysis of the endophytic fungi communities in seven parts of *Epidendrum* sp., we detected the colonization of the Epi910 isolate in all seven samples. Thus, it can be considered that the Epi910 isolate is an endophytic fungus of orchids. Using symbiotic germination, we observed that other isolates of *Coriolopsis strumosa* could also promote the seed germination of *Epidendrum* sp. This suggests that a germination-enhancing ability might be prevalent within certain *Coriolopsis strumosa* strains.

### 4.1. Isolation of Coriolopsis strumosa Strain Epi910

The Epi910 isolate was acquired from an asymbiotically germinated protocorm in a Petri dish, which was superior to other dishes for seed germination. The molecular identification result based on the ITS and nLSU sequences indicated that Epi910 belonged to *Coriolopsis strumosa*, a common forest white-rot fungus. We speculated that Epi910 might be an endophytic fungus present in the seeds rather than a result of the culturing environment.

Recent studies have revealed that certain microorganisms in seeds can promote seed germination. *Penicillium* from the seeds of *Phragmites australis* was strongly associated with an increase in the germination of seeds from Sandusky, USA [51]. Seed-borne fungal endophytes belonging to the genus *Epichlöe* assisted their host plants with growth promotion and stress mitigation [52,53]. *Cladosporium* isolated from seeds was demonstrated to have positive effects on germination and plant growth in *Zea mays* and American sweetgum seedlings [54,55]. Seed-borne endophytes in grass were considered to be good examples of mutualism [56]. Research on orchid endophytic fungi has mainly focused on mycorrhizal fungi from the roots and in situ germinated seeds; seed-borne fungi have not received extensive attention among orchid fungal endophytes.

In this study, we used the establishment of a symbiotic relationship between Epi910 and the seeds of *Epidendrum* sp., the formation of a typical intracellular OMF structure, and the promotion of seed germination and seedling formation as the basic standards to determine that the isolate was a germination-enhancing fungus.

### 4.2. Promoting Effect of White-Rot Fungi on Seed Germination

Orchids mainly associate with a polyphyletic group of fungi, collectively called rhizoctonia-like fungi (Ceratobasidiaceae, Sebacinales, or Tulasnellaceae). Certain orchid species target other fungal groups that differ from usual OMF, with differences in the phylogenetic position and/or ecological traits [57,58]. Several groups of saprotrophic fungi, for example *Mycena* [59], *Coprinellus* [60], and *Trametes* [61], can colonize the roots of orchids and promote their growth or can serve as mycorrhizal fungi. Recent research suggests that mycorrhizal fungi could be recruited from the endophytic fungi colonized in orchid ancestors [57].

Certain white-rot fungi exhibit functions similar to those of OMF or ectomycorrhizal fungi in their genomic characteristics. In Cantharellales, the white-rot fungus *Botryobasidium botryosum* and ORM (*Tulasnella calospora* and *Ceratobasidium* sp.) both possess large sets of enzymes that act on cellulose, hemicellulose, and pectin, including lytic polysaccharide monooxygenases (LPMOs) [62]. With an increase in research into the functions of OMF and the endophytic fungi of orchids, it should be possible to discover the similarities in genomes or gene expression levels among germination-enhancing fungi in orchids.

### 4.3. Spatial Distribution of Epi910 in Epidendrum sp.

Microbes can colonize different plant organs and prosper inside plant tissues [63]. In this study, we explored the fungi associated with different parts of *Epidendrum* sp., including the protocorm and seed.

A total of 30 common OTUs, including Epi910 (OTU6), were detected in seven parts of *Epidendrum* sp. The highest relative abundance was observed in the pericarp, followed by the seed and root. The relative abundance of OTU6 in the protocorm germinated in vitro significantly decreased and was close to that in the rachis, epiphytic root, and ovary. We speculated that Epi910 exists in the endophytic systems of *Epidendrum* sp. from the root to the flower organs and may be transmitted to the seed during seed maturation. This theory was based on the high relative abundance observed in the pericarp and seed. The relative abundance of Epi910 decreased to a level characteristic of an endophytic fungus with low abundance after the seed germinated into the protocorm.

## 5. Conclusions

A *Coriolopsis strumosa* strain (Epi910) was isolated from the asymbiotic germinated protocorm of *Epidendrum* sp. and identified as a germination-enhancing fungus. High-throughput sequencing of the endophytic fungal communities in different parts of *Epidendrum* sp. revealed that Epi910 was identical to a dominant OTU (OTU6), which was detected in all seven samples. The results indicated that there may be other endophytic fungi in addition to common orchid mycorrhizal fungi with germination-enhancing abilities and endophytic fungi in seeds may have a promoting effect on seed germination. Attention should be paid in future research to functional endophytic fungi in orchids, which could have an equally important ecological value as OMF in Orchidaceae plants.

## Data Availability

The original contributions presented in the study are included in the article/supplementary material, further inquiries can be directed to the corresponding author.

## Supplementary Materials

The following supporting information can be downloaded at https://www.mdpi.com/article/10.3390/microorganisms12061054/s1. Table S1: The relative abundance of shared OTUs for each sample; Table S2: Relative abundance of OTU6 (*C. strumosa*) in all seven samples.
